# Acute appendicitis caused by endometriosis: a case report

**DOI:** 10.1186/1752-1947-5-144

**Published:** 2011-04-11

**Authors:** Styliani Laskou, Theodossis S Papavramidis, Angeliki Cheva, Nick Michalopoulos, Charilaos Koulouris, Isaak Kesisoglou, Spiros Papavramidis

**Affiliations:** 1 Third Department of Surgery, AHEPA University Hospital, Aristotle University of Thessaloniki, Thessaloniki, Greece; 2 Department of Pathology, AHEPA University Hospital, Aristotle University of Thessaloniki, Thessaloniki, Greece

## Abstract

**Introduction:**

Endometriosis is a well-recognized gynecological condition in the reproductive age group. Surgical texts present the gynecological aspects of the disease in detail, but the published literature on unexpected manifestations, such as appendiceal disease, is inadequate. The presentation to general surgeons may be atypical and pose diagnostic difficulty. Thus, a definitive diagnosis is likely to be established only by the histological examination of a specimen.

**Case presentation:**

We report a case of endometriosis of the appendix in a 25-year-old Caucasian woman who presented with symptoms of acute appendicitis and was treated by appendectomy, which resulted in a good outcome.

**Conclusions:**

We discuss special aspects of acute appendicitis caused by endometriosis to elucidate the pathologic entity of this variant of acute appendicitis.

## Background

Endometriosis is the presence of endometrial glands and stroma outside the uterine cavity and musculature [1]. It affects 4% to 50% of women of reproductive age and results in pelvic pain in up to 50% of these patients [2]. The symptomatology of the disease is often related to the location of the lesions [3], and for that reason endometriosis of the gastrointestinal tract, although rare, may cause a wide spectrum of symptoms [4-6]. Appendiceal endometriosis not only may cause symptoms of acute appendicitis [7-10] but also is known to cause cyclic and chronic right lower quadrant pain [11], melena [12], lower intestinal hemorrhage [13], cecal intussusceptions [14,15] and intestinal perforation, especially during pregnancy [16].

Appendiceal endometriosis was first described in 1860 [17], while in 1951 Collins [12] reviewed a total of 150 cases in the literature. Four years afterward Collins further described more than 50,000 random pathologic assessments of the appendix and reported the prevalence of appendiceal endometriosis as 0.054% [18]. More recent studies, however, have reported the prevalence of appendiceal endometriosis to be around 0.8% [19].

We describe a case of a woman with appendiceal endometriosis that presented as acute appendicitis. We additionally discuss special aspects of the disease to elucidate this variant of acute appendicitis.

## Case presentation

A 25-year-old Caucasian woman was admitted to our hospital with a two-day history of lower quadrant abdominal pain. She had no fever, but she reported nausea, vomiting and anorexia. Her McBurney's point was positive with abdominal guarding and rigidity. She had no relevant gynaecological history.

**Figure 1:**
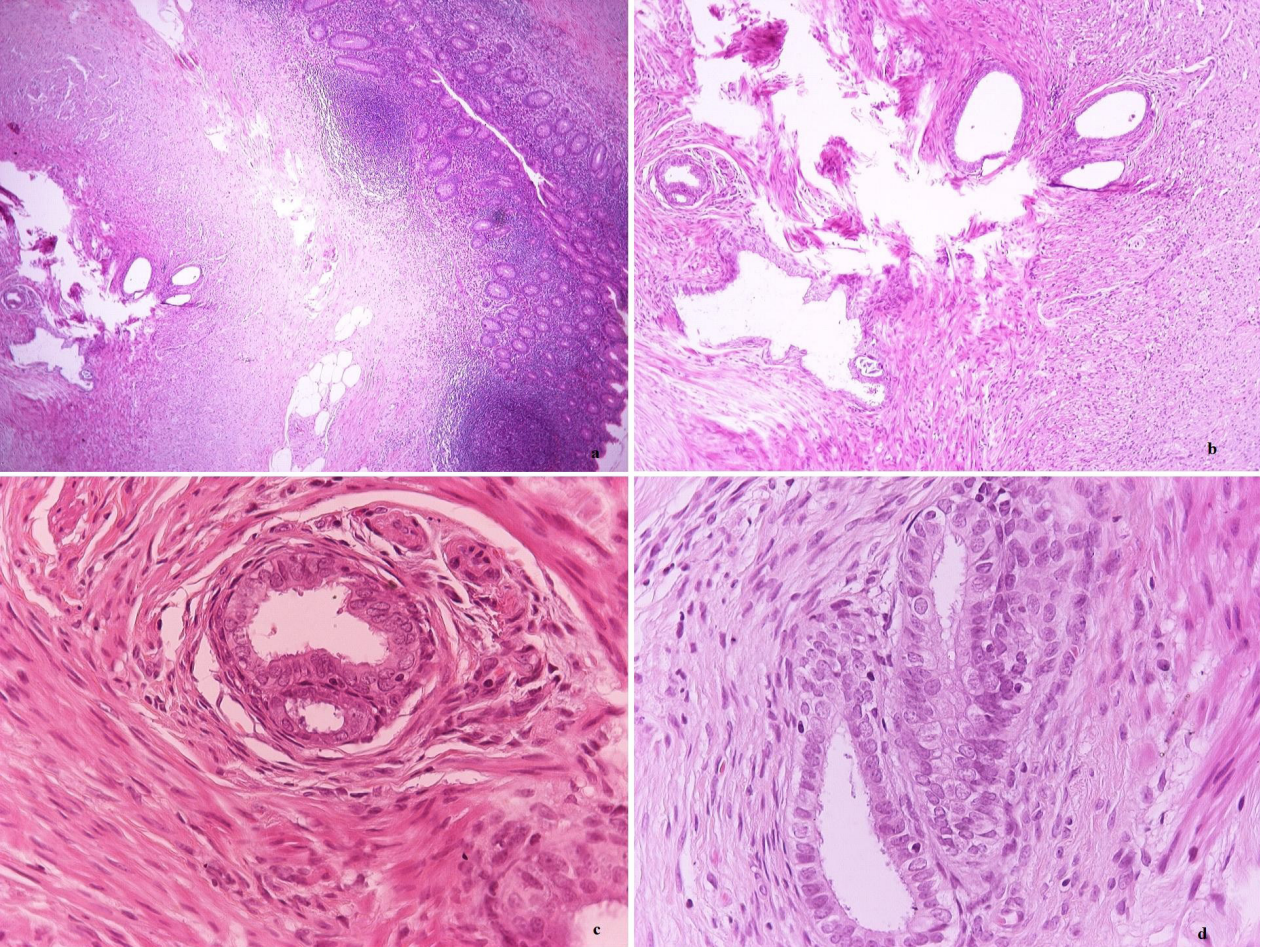
**Small nodules found in the wall of the appendix**. The endometrial glands are surrounded by endometrial stroma.

The patient's white blood cell count was 12,400/mm^3 ^with 83% neutrophils. Her urine analysis was normal, and her urine pregnancy test was negative. Acute appendicitis was diagnosed, and an appendectomy was performed. Intraoperatively, the appendix appeared mildly congested. The appendix measured 6.5 × 0.6 cm at the widest diameter. The pathological examination revealed small nodules found in the wall of the appendix. The patient's ectopic endometrial glands were surrounded by endometrial stroma (Figure 1). The pathology report led to the diagnosis of appendiceal endometriosis. Postoperatively, the patient recovered with no residual pain. Today, five years after the patient's appendectomy, her gynecologic anamnestic record remains clear and her follow-up with echograms has revealed no other sites of endometriosis.

## Discussion

When endometrial tissue is found outside its normal location, it is called "endometriosis". This condition is seen in 10% of women within their menstrual age. It is called "adenomyosis" or "internal endometriosis" when the endometrial tissue is found within the uterine muscles. External endometriosis is commonly found in the genital organs and the pelvic peritoneum [20].

The true prevalence of extragenital endometriosis is unknown because of a lack of large, well-defined case series. Case reports throughout the literature describe extragenital endometriosis in almost every organ and tissue in the body [21]. It may be seen in the gastrointestinal system, omentum, mesentery, liver, operation scars and, rarely, in the kidneys, lungs, central nervous system, skin and extremities [20-22]. Interestingly, one of the only sites where extragenital endometriosis has not been reported is the spleen [21]. With regard to the type of appendiceal endometriosis that we describe here, its incidence is thought to be low and is considered to be between 0.054% and 0.8% [18,19].

Several theories have been proposed to explain the pathogenesis of extragenital endometriosis [22]. The implantation or retrograde menstruation theory proposes that endometrial tissue from the uterus is transported in a retrograde fashion through the fallopian tubes [23]. The direct transplantation theory and the dissemination theory can explain extrapelvic endometriosis [24,25]. The coelomic metaplasia theory hypothesizes that the peritoneal cavity contains progenitor cells or cells capable of differentiating into endometrial tissue [26,27]. The induction theory suggests that sloughed endometrium produces substances to form endometriosis. The embryonic rest theory claims that a specific stimulus to a Müllerian origin cell nest produces endometriosis. The most recently developed theory is the cellular immunity theory, which suggests that alterations in cell-mediated and humoral immunity allow ectopic endometrial cells to proliferate [22].

Appendiceal endometriosis patients can be categorized into four groups in terms of symptomatology: (1) patients who present with acute appendicitis; (2) patients who present with appendix invagination; (3) patients manifesting atypical symptoms such as abdominal colic, nausea and melena; and (4) patients who are asymptomatic. These four patient groups are discussed in the subsections that follow.

Acute appendiceal inflammation can arise because of partial or complete luminal occlusion by the endometrioma [28]. Another mechanism suggested is that of endometrium hemorrhage within the seromuscular layer of appendix, which is followed by edema, obstruction and inflammation. Pain in the right lower abdominal quadrant is one of the most common symptoms, and one-third of those patients present with a typical appendiceal symptomatology [20]. The routine examination of a patient suspected of having acute appendicitis consists of a complete blood count and urine analysis. The most important diagnostic tool is still a physical examination, but use of imaging studies is increasing day-by-day. This is a result of the need for early diagnosis and treatment to achieve a lower perforation rate and fewer complications [29]. Leukocytosis with the predominance of polymorphonuclear leukocytes accompanies acute appendicitis in most cases, along with elevated C-reactive protein. In our patient, fever was absent, but there was an increase in leukocytes. Computed tomographic scans obtained to diagnose appendiceal endometriosis often show a distended, nonopacified appendix without inflammation [30].

Along with foreign bodies, inflammation, polyps and neoplasia, endometriosis should be considered as a possible cause of appendiceal invagination [20]. Appendiceal intussusception is uncommon (incidence of 0.01%). Endometrial involvement of the appendix is usually accompanied by chronic fibrosis, inflammation and hyperplasia or hypertrophy of the muscularis propria. This hypertrophic segment serves as a lead point for hyperperistalsis, hence making it prone to invagination, particularly when combined with a fully mobile appendix that has a wide proximal lumen and a fat-free mesoappendix. Patients often present with weeks to months of intermittent abdominal pain, nausea, vomiting, melena (or "currant jelly stool"), fever or constipation [31]. Occasionally, patients are asymptomatic. The radiographic findings are generally normal unless a small-bowel obstruction exists. Sonography may identify the classic target lesion, or "donut sign," associated with intussusception [32]. Computed tomographic abdominal scans may demonstrate a soft tissue mass in the region of the cecum, although it may not lead to the diagnosis [28].

Patients who fall within these groups do not manifest signs of either appendicitis or ileus. These two groups are usually diagnosed incidentally [20].

Appendiceal endometriosis is often seen in patients with ovarian endometriosis. Appendectomies were performed in 65 of 125 patients with ovarian endometriosis who underwent various operations because of infertility. Thirteen of the appendectomy pathological examinations revealed appendiceal endometriosis. This result has led to a discussion whether to perform elective appendectomies in patients who have undergone gynecological operations because of endometriosis [20]. Moreover, endometriosis of the appendix is reported to have a high incidence of association with leiomyoma of the uterus and menstrual abnormalities [8]. Some authors have even reported the cases of endometriosis patients with symptoms of abdominal pain with menstruation. However, our patient had no history of these abnormalities, and her symptoms did not coincide with menstruation.

Appendiceal endometriosis is diagnosed pathologically. Glandular tissue, endometrial stroma and hemorrhage are typical examinations conducted in patients with endometriosis [20]. About half of endometriosis of the appendix involves the body and half involves the tip of the appendix. Muscular and seromuscular involvement occurs in two-thirds of patients, while the serosal surface is involved in only one-third of patients. The mucosa is not involved, but Langman *et al*. [33] found that the submucosa was involved in one-third of patients with endometriosis of the appendix. In their series, the endometriotic foci were also found in the muscle, serosa and subserosa. There was no correlation between the location of the endometriotic foci and the patients' symptoms [33]. Therefore, mucosal or submucosal endometriosis is much more likely to mimic primary inflammatory diseases such as Crohn's disease, infectious or ischemic enteritis or colitis, or mucosal prolapse than endometriosis of the outer bowel wall [31]. Our patient is categorized in the typical form of appendiceal endometriosis, since small nodules were present in the wall of the appendix while the endometrial glands were surrounded by endometrial stroma.

The treatment consists mainly of surgery and hormone therapy. The treatment tends to be determined by the age of the patient and the degree of the patient's symptoms. Thus, the extent of resection should be appropriate. Intraoperative investigations usually result in an accurate diagnosis of endometriosis with minimal resection. A gynecological assessment should be performed to determine the extent of endometriosis, and postoperative follow-up is mandatory for appendiceal endometriosis. In our patient, the postoperative gynecological examination did not reveal any other endometriotic lesions [30]. Laparoscopic appendectomy is now commonly performed for appendicitis. Laparoscopic surgery is useful for women with chronic abdominal pain caused by endometriosis, ovarian cysts, adhesions and hernias. Laparoscopy enables the exploration of the total peritoneal cavity and the selection of the appropriate method for a definitive diagnosis. Medical treatments for endometriosis are secondary. Appendiceal endometriosis appears to be an incidental finding and one that is not clinically important [31].

## Conclusion

Appendiceal endometriosis is rare, and its preoperative diagnosis is difficult. However, it should be included in the differential diagnosis of acute abdominal pain, especially when women of childbearing age present with clinical symptoms of acute appendicitis but no evidence is observed on imaging studies. Laparoscopy is useful for the diagnosis, and appendectomy relieves the acute symptoms.

## Consent

Written informed consent was obtained from the patient for publication of this case report and accompanying images. A copy of the written consent is available for review by the Editor-in-Chief of this journal.

## Competing interests

The authors declare that they have no competing interests.

## Authors' contributions

SL analyzed and interpreted the patient data and drafted the manuscript. TSP received the patient in the outpatient department. NM and CK received the patient in the outpatient department, served as auxiliary surgeons and drafted the manuscript. AC performed the pathological examination and was a major contributor in writing the manuscript. IK was the principal surgeon and drafted the manuscript. SP was responsible for the overall treatment of the patient and corrected the manuscript. All authors read and approved the final manuscript.
